# Investigation of the Effects and Mechanisms of Mai Tong Formula on Lower Limb Macroangiopathy in a Spontaneous Diabetic Rat Model

**DOI:** 10.1155/2016/8076796

**Published:** 2016-11-22

**Authors:** Guangming Gong, Haipo Yuan, Ya Liu, Luguang Qi

**Affiliations:** Endocrinology Department, Teaching Hospital of Chengdu University of Traditional Chinese Medicine, Chengdu 610075, China

## Abstract

A new Chinese herbal formula called Mai Tong Formulae (MTF) has recently been used to treat lower limb macroangiopathy in type 2 diabetes mellitus (T2DM) patients. In this study, we investigated the effect of MTF on lower limb macroangiopathy in a spontaneous diabetic rat model (GK rats). We found that MTF treatment significantly reduced serum fasting blood glucose (FBG), triglycerides (TG), total cholesterol (TC), IL6, and VEGF and increased serum insulin in this model. Histological and ultrastructural observations showed that MTF treatment significantly reduced vascular endothelial cell shedding and improved endothelium injuries. We further detect proteome alteration following MTF treatment. 25 differential proteins (DPs) abnormally expressed in GK rats were normalized by MTF treatment. These DPs significantly are enriched in biological processes and pathways that regulate muscle contraction and cGMP-PKG signaling pathway and so on. Additional protein-protein interaction (PPI) network analyses of the DPs showed that Fasn and Prkar2a are involved in the AMPK signaling pathway, and Gnas, Myh11, and Myh6 are involved in vascular smooth muscle contraction; these 5 DPs were validated by Western blotting. These results indicate that MTF treatment effectively treats lower limb macroangiopathy by regulating key proteins involved in AMPK signaling pathway and vascular smooth muscle contraction.

## 1. Introduction

The prevalence of diabetes mellitus is increasing dramatically worldwide. Reports showed that, in 2015, over 8% of adult people worldwide suffer from type 2 diabetes mellitus (T2DM) (International Diabetes Federation, 2015) [1]. Lower limb macroangiopathy is a major complication T2DM which is caused by various pathogenic factors, including blood glucose levels, peripheral neuropathy, oxidative stress, and ischemia [2, 3]. Lower limb macroangiopathy can develop into lower limb ulcers, which decrease quality of life and contribute to high morbidity, mortality, and healthcare costs [4]. Currently, there is no medication to cure T2DM or macroangiopathy complications. In Western medicine, some blood glucose-lowering drugs, such as rosiglitazone and glibenclamide, have been used to alleviate the symptoms of T2DM but significant side effects and drug resistance exist [5]. Therefore, the development of novel longer-lasting, targeted therapeutics is urgently needed.

In China, traditional herbal formulas have been used for centuries to treat T2DM [6, 7]. Many herbal extracts can reduce blood glucose and improve complications associated with T2DM [8–11], and, compared to Western medications, many Chinese herbs have fewer side effects [12]. However, the complexity and various actions of herbal components have limited their application worldwide.

Recently, a new herbal formula called Mai Tong Formula (MTF) has been used to treat lower limb macroangiopathy and diabetic foot ulcers (DFU) in T2DM patients and has a curative effect on hyperglycemia, atherosclerosis, nephropathy, and inflammation [13]. However, the effect of MTF treatment on lower limb macroangiopathy has not been evaluated systematically, and no studies have explored the molecular mechanism of this herbal formula. Herein, we aim to explore the effect and molecular mechanism of MTF using a spontaneous diabetic rat model (GK rats). First, we evaluated the effect of MTF by measuring several blood biochemical indicators and histological observation; then, we employed quantitative proteomic assays using isobaric tags for relative and absolute quantitation (iTRAQ), combined with high performance liquid chromatography-tandem mass spectrometry (HPLC-MS/MS), to detect proteome alteration from MTF treatment. Additional bioinformatics analyses were used to analyze the differential proteins (DPs) to investigate the key pathways underlying the mechanism of MTF treatment.

## 2. Materials and Methods

### 2.1. Animal Model and MTF Preparation

MTF is composed of dozens of herbs, including Huangqi (Radix Astragali), Sangshen (Fructus Mori), Danggui (*Angelica sinensis*), Danshen (*Salvia miltiorrhiza* Bge.), Zexie (*Alisma plantago-aquatica *Linn.), and Yinhuateng (*Lonicera japonica* Thunb.). All herbs were decocted with water, filtered, and brought to a final concentration of 1.0 g/mL.

Experimental protocols were approved by the Experimental Animal Care and Ethics Committees of the Teaching Hospital of Chengdu University of Traditional Chinese Medicine. Seven normal rats and 14 spontaneous diabetic rat models (Goto-kakizaki rats, GK rats) were purchased from SLRC Laboratory Animal Co., Ltd. (Shanghai, China). All rats were 8-week-old females with a body weight between 150 g and 210 g (certification number: SCXK (hu) 2003-0003). Seven normal rats were used as a control group and were given 2 mL intragastric saline vehicle (0.9%) once a day for 12 weeks. 14 GK rats were randomized and divided into two groups: (1) the model group (*n* = 7) was given 2 mL of intragastric saline vehicle (0.9%) once a day for 12 weeks; (2) the MTF group (*n* = 7) was given 29 g/kg MTF decoction once a day for 12 weeks. All rats were anesthetized and sacrificed under the experimental protocols described above and all efforts were made to minimize suffering.

### 2.2. Blood Indicators Examination

We removed the rats' tails and obtained samples to measure fasting blood glucose (FBG), blood triglycerides (TG), and blood total cholesterol (TC) levels in the 12th week of testing (AU5800, Beckman Coulter, USA). Serum insulin was measured by radioimmunoassay (HTA Co., Ltd., Beijing, China, number 2013009) using 2 mL inner canthus blood. Vascular endothelial growth factor (VEGF) and serum interleukin 6 (IL6) were measured by double-antibody sandwich ELISA (BOSTER Inc., Wuhan, China, number 2013006) using 2 mL inner canthus blood.

### 2.3. Pathologic Histology

For histological observation, rat femoral arteries were excised and fixed in 10% neutral formalin paraffin-embedded after dehydration; then, sections of tissue were stained with hematoxylin and eosin and images were obtained by light microscopy (Olympus, Tokyo, Japan). For ultrastructural observation, the femoral arteries were excised and fixed in 3% glutaraldehyde (4°C), dehydrated, embedded, and cut into semithin sections for optical localization; ultrathin sections were stained and visualized by electron microscopy (JEM-1010 (HC), JEOL).

### 2.4. Proteomic Analysis

0.7 g of femoral arteries from 7 rats of each group (0.1 g per rat) was collected for protein extraction; then, protein (100 *μ*g) was digested with trypsin for 12 h at 37 °C (protein/enzyme = 100/3.3). After iTRAQ (AB Science) labeling, equal amounts of labeled peptides from each group were mixed and resolved into 15 fractions by high performance liquid chromatography (HPLC), followed by Q Exactive mass spectrometry (Thermo Fisher Scientific). The resulting MS/MS data were qualitatively and quantitatively analyzed by Mascot 2.3.01 with the following parameters: protein identification using the nonredundant International Protein Index rat protein database (version 3.72) and full trypsin digest with a maximum of 1 missed cleavage. Peptide tol. and MS/MS tol. were 0.05 Da. Scaffold software was used to identify the differential proteins (Dps). Proteins with *P* < 0.05 fold change higher than 1.2 or lower than 0.833 were DPs.

### 2.5. Western Blotting Analysis

Cell lysates were separated by SDS-PAGE in 8% Tris-glycine gels (Invitrogen Life Technologies, Carlsbad, CA, USA) and transferred to a nitrocellulose membrane. Blots were probed with specific antibodies [diluted with 5% bovine serum albumin (BSA) to 1 : 1000]. Membranes were probed with horseradish peroxidase-labeled anti-rabbit secondary antibody (diluted with 5% BSA to 1 : 1000; Cell Signaling Technology, Inc., Danvers, MA, USA). Antibody binding was detected by using an enhanced chemiluminescence detection kit (Amersham International PLC, Buckinghamshire, UK).

### 2.6. Data Preprocessing

The data are presented as mean ± standard deviation. Statistical comparisons among the three experimental groups were made using unpaired Student's *t*-tests. The GO and KEGG pathway enrichment analysis of DPs were performed using the Database for Annotation, Visualization, and Integrated Discovery (DAVID) [14]. The protein-protein interaction (PPI) networks were constructed using the String 10.0 database [15].

## 3. Results

### 3.1. Blood Indicators Examination and Pathologic Histology

From 0 to 12 weeks, compared with the control group, the rats in the model group were inactive, withered, and lusterless and had sparse fur, experienced diarrhea, and consumed more food and water. These symptoms in the MTF group were significantly alleviated.

**Figure 1:**
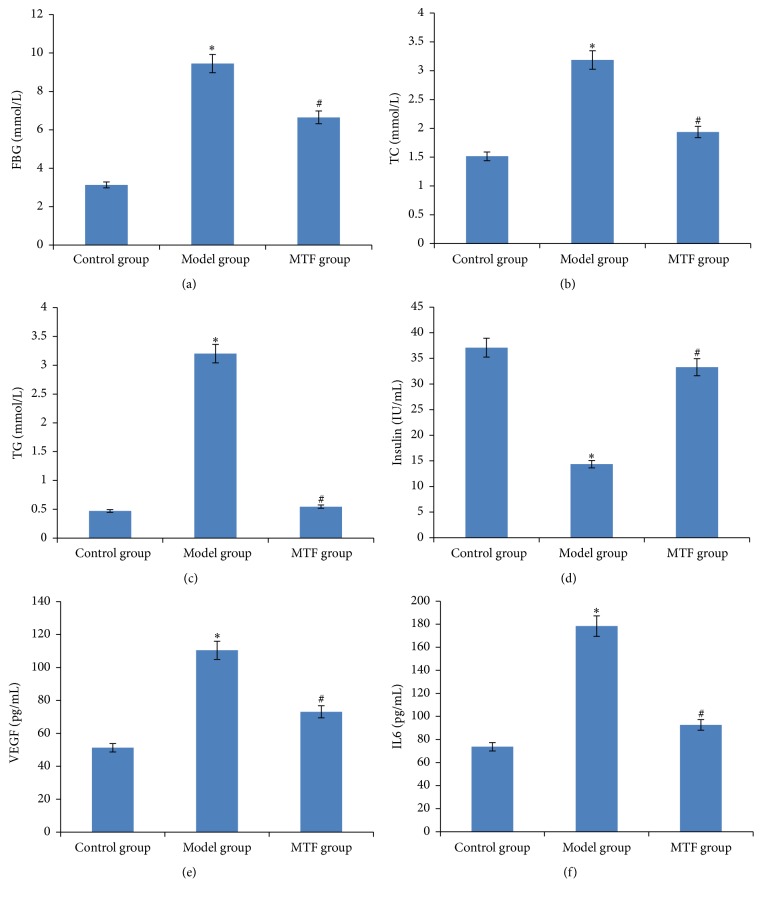
Blood indicators across three groups. (a) Level of FBG in blood; (b) level of TC in blood; (c) level of TG in blood; (d) level of insulin in blood; (e) level of VEGF in blood; (f) level of IL6 in blood. ^*∗*^Significant difference between the control and model group (*P* < 0.01). ^#^Significant difference between the model and MTF group (*P* < 0.01).

We detected FBG, TG, and TC for all three experimental groups in the 12th week. As the results show in Figures 1(a), 1(b), and 1(c), the levels of FBG, TG, and TC in the model group were significantly higher than in the control group (*P* < 0.01), and, compared to model group, FBG, TG, and TC levels in the MTF group significantly improved (*P* < 0.01). As shown in Figure 1(d), serum insulin levels in the model group were significantly lower than in the control group (*P* < 0.01), and, compared to the model group, serum insulin levels in the MTF group were significantly increased (*P* < 0.01). As shown in Figures 1(e) and 1(f), serum VEGF and IL6 levels were significantly higher than in the control group (*P* < 0.01), and, compared to the model group, serum VEGF and IL6 levels were significantly reduced (*P* < 0.01).

**Figure 2:**
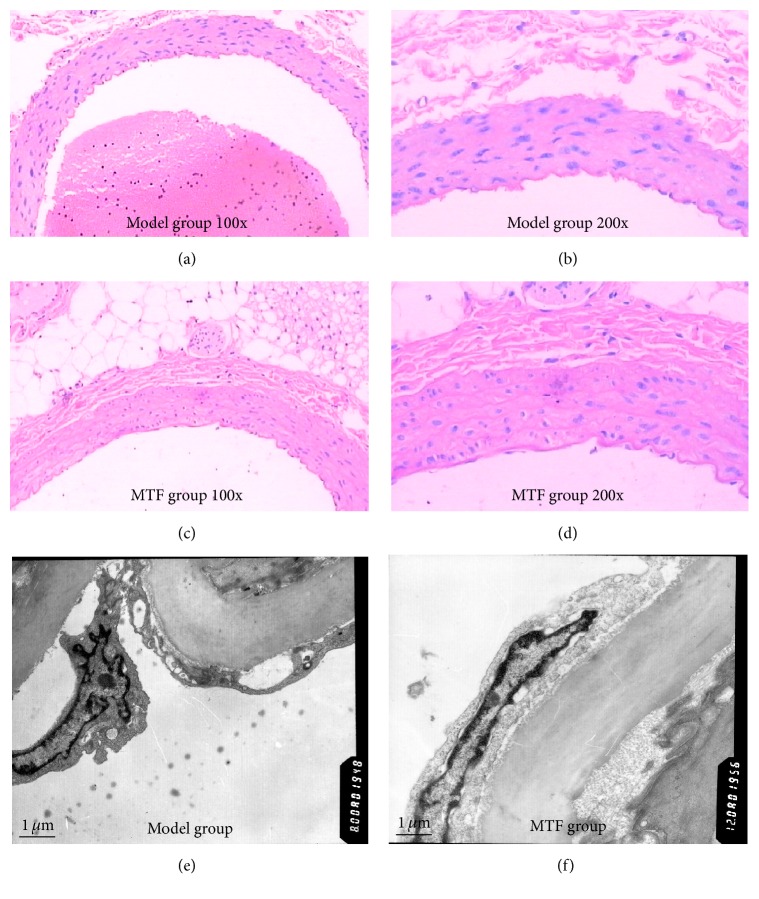
Histomorphological and ultrastructural observations of the femoral artery. (a) Histomorphological observation of model group (magnification ×100); (b) histomorphological observation of model group (magnification ×200); (c) histomorphological observation of MTF group (magnification ×100); (d) histomorphological observation of MTF group (magnification ×200); (e) ultrastructural observation of model group; (f) ultrastructural observation of MTF group.

Histological images from the model and MTF groups are shown in Figures 2(a), 2(b), 2(c), and 2(d). In the model group, there was a large amount of vascular endothelial cell shedding, and the area of exfoliated endothelial cells was greater than 80%; however, in the MTF group, the exfoliated endothelial cells were distributed locally and the area was lower than 30%. The internal elastic lamina and vascular adventitia did show distinct changes in either group. The results of ultrastructural observation are shown in Figures 2(e) and 2(f). In the model group, endothelial cell shedding, nuclear atypia, and chromatin edge accumulation were seen. Compared with the model group, the ultrastructure of endothelial cells in the treatment group was more complete.

### 3.2. Proteomics Analysis of the Three Groups

**Figure 3:**
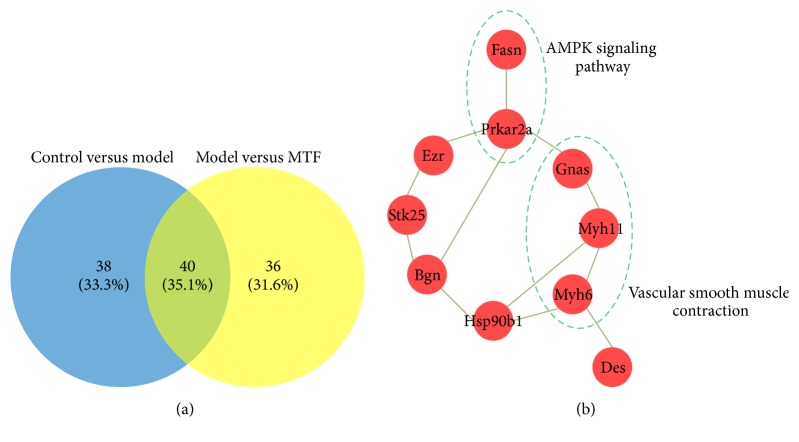
Venny plot of DPs between each group and the PPI network of the DPs normalized by MTF treatment. (a) Venny plot of DPs between each group (control versus model and model versus MTF); (b) PPI network of the DPs normalized by MTF treatment. Line: interaction; red node: DPs.

To explore the molecular mechanism of MTF, the femoral arteries of the three groups were collected for proteomics analysis using an iTRAQ approach. A total of 764 proteins were identified (see Table S1 in Supplementary Material available online at http://dx.doi.org/10.1155/2016/8076796); there were 78 DPs between the control and model groups and 76 DPs between the model and MTF groups (see Tables S2 and S3). As shown in Figure 3(a), 40 overlapping DPs were found between the two DP comparisons (control versus model and model versus MTF). Deep analysis of these 40 overlapping DPs revealed that 25 DPs were abnormally expressed in the model group and normalized in the MTF group (named MTF-normalized DPs); 11 DPs were upregulated in the model group and downregulated in the MTF group; and 14 DPs were downregulated in the model group and upregulated in the MTF group (Table 1). We further investigated the related biological functions of the MTF-normalized DPs by using the David database. As shown in Table 2, the 25 DPs were significantly enriched in metabolic processes, cellular chemical homeostasis, muscle contraction, the cGMP-PKG signaling pathway, and endocrine and other factor-regulated calcium reabsorption pathways.

### 3.3. PPI Network of the DPs

To investigate the relationship between the 25 MTF-normalized DPs, we constructed a PPI network using a 10.0 database. As shown in Figure 3(b), the PPI network included 10 DPs and 12 interactions between them. In this PPI network, Fasn and Prkar2a and their interaction were found to be involved in the AMPK signaling pathway; these two DPs were downregulated in the model group and upregulated in the MTF group. Gnas, Myh11, and Myh6 and their interactions were also found to be involved in vascular smooth muscle contraction; these three DPs were upregulated in the model group and downregulated in the MTF group.

### 3.4. Validation of Representative DPs by Western Blotting

**Figure 4:**
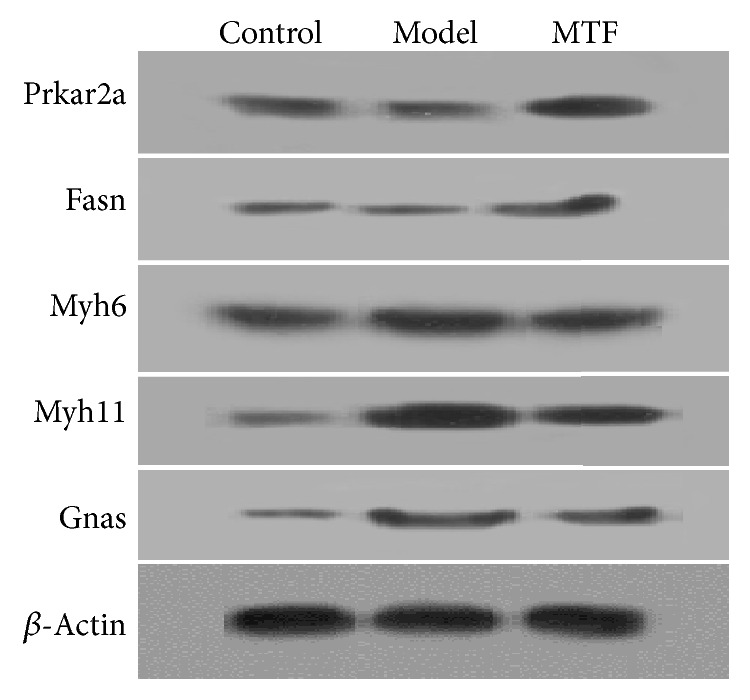
Western blots of the five DPs (Prkar2a, Fasn, Gnas, Myh11, and Myh6).

To verify proteomic analysis data, five DPs (Fasn, Prkar2a, Gnas, Myh11, and Myh6) were validated using Western blotting. As shown in Figure 4, the expression of all five DPs was consistent with the iTRAQ data. Fasn and Prkar2a were downregulated in the model group and upregulated in the MTF group. Gnas, Myh11, and Myh6 were upregulated in the model group and downregulated in the MTF group.

## 4. Discussion

Lower limb macroangiopathy is a major complication associated with diabetes mellitus and has been shown to precede amputation in up to 90% of cases [16]. Current studies have indicated that continuous metabolic and chronic hemodynamic alterations in the blood of patients with T2DM could damage the endothelium, resulting in structural and functional changes and leading to thickening of the basement membrane and sclerosis of vascular walls, which can cause lower limb macroangiopathy [17, 18]. Many biological processes are involved in the pathogenesis of macroangiopathy, including oxidative stress, collagen deposition, angiogenesis, ECM remodeling, and vascular remodeling [19–21]. Improvement of these biological processes may be fundamental to treating lower limb macroangiopathy in T2DM.

In this study, we evaluated the efficacy and the molecular mechanism underlying MTF treatment. Our results showed that MTF treatment significantly reduced serum FBG, TG, and TC levels and significantly increased serum insulin levels in GK rats. We also found that MTF treatment significantly reduced levels of VEGF, which is associated with atherosclerosis, vascular ischemia, hypoxia, and AGE accumulation in T2DM [22]. This reduction in VEGF indicates that MTF treatment may address antiatherosclerosis. Moreover, we found that MTF treatment also significantly reduced IL6 levels in GK rats, suggesting anti-inflammation effects [23], since IL6 is associated with vascular smooth muscle contraction in rat models of diabetes [24]. Based on visual inspections, we found a significant reduction in vascular endothelial cell shedding and improvement in endothelium injuries. Ultrastructural observation showed that MTF treatment also significantly improved necrosis in vascular endothelial cells. Taken together, these results indicate that MTF alleviates angiogenesis and affects vascular remodeling.

To characterize the molecular mechanism of MTF treatment, a proteomics analysis was used to explore multitarget characteristics. Abnormal expression of 25 DPs was normalized by MTF treatment in the GK rats. Functional enrichment analysis showed that these DPs were significantly involved in cellular chemical homeostasis, muscle contraction, cGMP-PKG signaling pathways, and endocrine and other factor-regulated calcium reabsorption, all of which are significantly associated with T2DM [25–31]. Moreover, we constructed a PPI network of these DPs, and, in this PPI network, Fasn and Prkar2a and their interaction were found to be involved in the AMPK signaling pathway. These 2 proteins have been proven to play key roles in macroangiopathy in T2DM. Fasn is a fatty-acid synthase, which may target endothelial nitric-oxide synthase (eNOS) in the plasma membrane by adding palmitate to eNOS; in this scenario, eNOS could generate NO in vascular tissue, and, in its absence, NO could decrease vasodilatation effectively leading to limb/nerve ischemia and macroangiopathy in T2DM [29]. Prkar2a is known to be a key upstream regulatory factor for Fasn [30, 32]. Three proteins (Gnas, Myh11, and Myh6) were found to be involved in vascular smooth muscle contraction, which is associated with vascular ischemia and poor local blood flow in macroangiopathy in T2DM [24]. These results indicate that MTF treatment could significantly improve vascular smooth muscle contraction.

**Figure 5:**
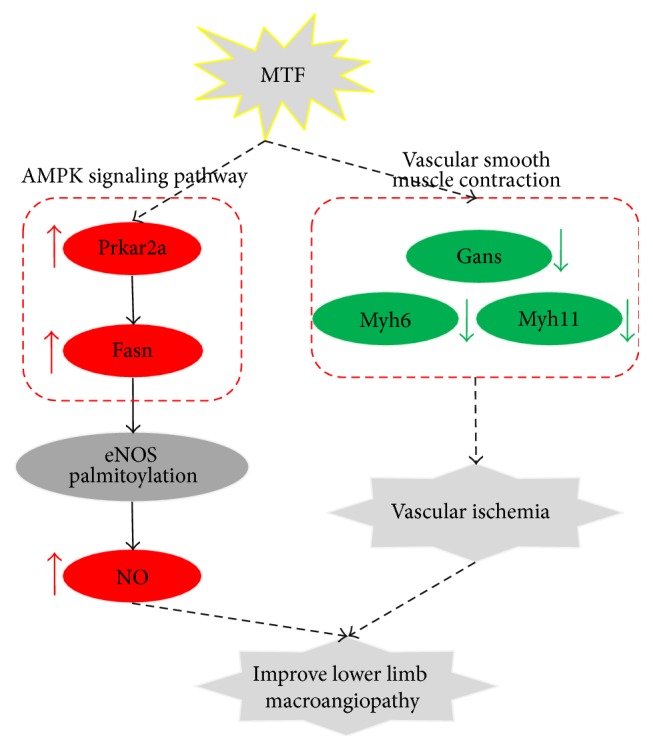
Mechanism of MTF treatment for lower limb macroangiopathy in T2DM.

T2DM is a complex disease that involves protein imbalance and the disturbance of multiple biological pathways. This study has shown that MTF can normalize many proteins and corresponding biological processes/pathways associated with T2DM. In accordance with our experimental results, we speculate a mechanistic pathway in vascular tissue that could be affected by MTF treatment (Figure 5). In this pathway, MTF could upregulate the expression of Prkar2a and Fasn, leading to eNOS palmitoylation, which would increase NO concentration and finally improve macroangiopathy. In addition, MTF could downregulate the expression of Gnas, Myh11, and Myh6 (which are associated with vascular ischemia), thus leading to improvement in macroangiopathy.

## Supplementary Material

All proteins and DPs identified by iTRAQ approach.

## Figures and Tables

**Table 1:** DPs normalized by MTF treatment.

#	Accession number	Symbol	Molecular weight	Ratio (model/control)	Ratio (MTF/model)
Up expressed in model then down expressed in MTF	IPI00189809	Myh6	224 kDa	1.32	0.57
	IPI00200352	Crip2	23 kDa	1.23	0.76
	IPI00869592	Mylk	217 kDa	1.23	0.81
	IPI00208061	Atp1b3	32 kDa	1.41	0.66
	IPI00199872	Gnas	46 kDa	1.41	0.71
	IPI00230787	Car2	29 kDa	1.23	0.81
	IPI00231662	Cyb5r3	34 kDa	1.62	0.54
	IPI00231968	Anxa4	36 kDa	1.23	0.76
	IPI00764167	myh11	228 kDa	1.32	0.81
	IPI00390595	Stk25	48 kDa	1.41	0.76
	IPI00421517	Des	53 kDa	1.32	0.81

Down expressed in model then up expressed in MTF	IPI00191090	Bgn	42 kDa	0.76	1.32
	IPI00200661	Fasn	273 kDa	0.62	1.52
	IPI00205332	Etfa	35 kDa	0.81	1.23
	IPI00213036	C4a	192 kDa	0.71	1.23
	IPI00231139	Tkt	71 kDa	0.41	2.46
	IPI00231368	Txn1	12 kDa	0.81	1.23
	IPI00326305	Atp1a1	113 kDa	0.44	1.52
	IPI00365985	Hsp90b1	93 kDa	0.71	1.32
	IPI00421539	Aco2	85 kDa	0.47	1.41
	IPI00470254	Ezr	69 kDa	0.81	1.23
	IPI00470288	Ckb	43 kDa	0.71	1.32
	IPI00480639	C3	186 kDa	0.81	1.23
	IPI00768626	Cdh5	87 kDa	0.71	1.52
	IPI00196684	Prkar2a	46 kDa	0.57	1.32

**Table 2:** GO and pathway enrichment analysis of the DPs normalized by MTF treatment database.

Database	Description	Protein number	*P*
Gene ontology	Metabolic process	16	5.59*E* − 04
	Single-organism metabolic process	11	2.76*E* − 03
	Small molecule metabolic process	8	4.84*E* − 03
	Regulation of sodium ion transport	3	2.09*E* − 02
	Cellular monovalent inorganic cation homeostasis	3	2.09*E* − 02
	Cellular potassium ion homeostasis	2	2.09*E* − 02
	Sodium ion export from cell	2	2.09*E* − 02
	Chemical homeostasis	6	2.09*E* − 02
	Cellular chemical homeostasis	5	2.09*E* − 02
	Membrane repolarization	2	2.09*E* − 02
	Inorganic ion homeostasis	5	2.09*E* − 02
	Positive regulation of striated muscle contraction	2	2.50*E* − 02
	Regulation of biological quality	8	2.50*E* − 02
	Cellular sodium ion homeostasis	2	3.38*E* − 02
	Response to organic substance	8	3.38*E* − 02
	Muscle contraction	3	4.78*E* − 02

KEGG pathway	Gastric acid secretion	5	2.44*E* − 06
	Proximal tubule bicarbonate reclamation	3	2.47*E* − 04
	Thyroid hormone synthesis	3	4.23*E* − 03
	Bile secretion	3	4.23*E* − 03
	Cardiac muscle contraction	3	4.50*E* − 03
	Pancreatic secretion	3	6.79*E* − 03
	Thyroid hormone signaling pathway	3	1.03*E* − 02
	Adrenergic signaling in cardiomyocytes	3	1.61*E* − 02
	cGMP-PKG signaling pathway	3	2.20*E* − 02
	Aldosterone-regulated sodium reabsorption	2	2.24*E* − 02
	Carbohydrate digestion and absorption	2	2.24*E* − 02
	Endocrine and other factor-regulated calcium reabsorption	2	2.50*E* − 02
	Mineral absorption	2	2.50*E* − 02
